# Sleeplessness

**Published:** 1889-10

**Authors:** 


### SLEEPLESSNESS.

The best remedy for sleeplessness is to wet half a towel, apply it to the back of the neck, pressing it up towards the base of the brain, and fasten the dry half of the towel over, so as to prevent too rapid exhalation. The effect is prompt and pleasant, cooling the brain, and bringing on a sweet slumber. Warm water is better than cold. To all suffering from overwork, excitement, or anxiety, this remedy will prove a boon.

What can missionaries in Africa expect to accomplish when Christian Nations continue to supply whiskey and rum in unlimited quantities? The statistics show that during a single year there were shipped to the west coast of Africa from Germany 7,136,236 gallons of rum, and from Great Britain, 601,328 gallons; from the United States whiskey and rum to the amount of 922,412 gallons. All the churches in the three nations could not overcome the devils inclosed in these shipments.

Some of the most successful and popular patent nostrums are, upon analysis, found to be wholly inert, except as they are made potent by an attractive name or reputed efficiency. A recent analysis of one of the most popular Russian patent medicines, to which most remarkable virtues have been ascribed, showed the only constituent to be water from the river Neva.—*Good Health.*

Every minute of the day seventy human beings are brought into existence and sixty-seven are removed, says a writer. The population of the world is steadily increasing at the rate of three per minute, of 4,320 per day, more than 1,500,000 per year. Just think of the yearly increase of man being equal to the entire population of the State of Iowa. Where do they all go? The home of the human race, so far as we able to learn, was in Asia, and from there all the nations have come. The rapid increase of population in the United States shows the tendency of the race to scatter and seek new fields.

The Intermarriage of Cousins.—The Legislature of Illinois has passed a law making the intermarriage of cousins a penal offense. This is an unwise law, first, because it interferes unduly with personal rights, and next, because it is uncalled for. The marriage of cousins who are of healthy family and physique, and especially if they are of different temperaments, is quite free from danger.—*Medical Record.*

A Cure for Rheumatism.—He who is able to allay human suffering without injury to the system is a public benefactor, and he who can produce a complete cure, by striking at the root of the disease and permanently eradicating it, is so in a much fuller degree. Of the latter class is Charles Dennin, the popular Pharmacist, at his old established stand, corner of Court Street and First Place, Brooklyn, compiler of Dennin's Certain Cure for Rheumatism and Gout, which has acquired a wide reputation for its manifold cures of these ever painful diseases. There is no mistake about it, as hundreds of the afflicted have testified.

